# Assessment of the Therapeutic Effect of Total Glucosides of Peony for Juvenile Idiopathic Arthritis: A Systematic Review and Meta-Analysis

**DOI:** 10.1155/2016/8292486

**Published:** 2016-07-25

**Authors:** Yongsong Cai, Qiling Yuan, Ke Xu, Jialin Zhu, Yuanbo Li, Xiaoqing Wu, Le Yang, Yusheng Qiu, Peng Xu

**Affiliations:** ^1^Department of Orthopaedics, The First Affiliated Hospital, Xi'an Jiaotong University Health Science Center, Xi'an 710061, China; ^2^Department of Joint Surgery, Xi'an Hong Hui Hospital, Xi'an Jiaotong University Health Science Center, Xi'an 710054, China

## Abstract

Juvenile idiopathic arthritis (JIA) is the most common rheumatic disease in children; some clinical trials have reported the effects of total glucosides of peony (TGP) in the treatment of JIA. However, no systematic review has yet been conducted. In this study, we assessed the efficacy and safety in patients with JIA enrolled in randomized controlled trials (RCTs) of TGP. We extracted data for studies searched from 8 electronic databases that were searched and also evaluated the methodological quality of the included studies. We assessed the following outcome measures: overall response rate, pain, tender joint count (TJC), swollen joint count (SJC), duration of morning stiffness (DMS), grip strength (GS), rheumatoid factor (RF), erythrocyte sedimentation rate (ESR), C-reactive protein (CRP), and adverse effects (AEs) in short term (4–8 weeks), intermediate term (9–26 weeks), and long term (>26 weeks). The final analysis showed that TGP acted as a unique nonbiologic disease-modifying antirheumatic drug (nonbiologic DMARD), and its therapeutic effects were safe and efficacious for the treatment of JIA with few AEs. However, more high-quality RCTs are needed to confirm these therapeutic effects.

## 1. Introduction

Juvenile idiopathic arthritis (JIA), defined as unexplained joint swelling present in children under 16 years of age that persists for at least 6 weeks, is the most common rheumatic disease in children [1]. The worldwide prevalence of JIA is 7–400/100000 [2]. Immune dysfunction is considered to be the key pathogenesis of the condition, but the etiology remains unclear [3]. The goal of medical management in JIA includes maximizing the protection of children's daily functional activities, relief of pain, prevention or reduction of organ damage, and minimizing drug toxicity [4]. The treatment options for JIA include nonsteroidal anti-inflammatory drugs (NSAIDs), corticosteroids, nonbiologic disease-modifying antirheumatic drugs (nonbiologic DMARDs), and biologic disease-modifying antirheumatic drugs (biologic DMARDs) (including TNF-*α* inhibitors, IL-1 inhibitors, IL-6 inhibitors, and other biologic compounds). The successful development of biologic DMARDs has provided a more efficient method for treating JIA [5].

*Paeonia lactiflora* Pallas, also named Chinese Peony, is a Chinese traditional medicine. In China, Korea, and Japan, the decoction of its dried root without the bark has been used in the treatment of rheumatoid arthritis (RA) for centuries and was approved to enter the market as a nonbiologic DMARD by the State Food and Drug Administration of China in 1998. A water or ethanol extract of the root, also known as total glucosides of peony (TGP), contains more than 15 components, of which paeoniflorin is the major active ingredient [6]. In recent years, some studies have demonstrated anti-inflammatory [7–9], immunomodulatory [10–12], hepatoprotective [13–18], and analgesic effects of TGP [19, 20] both in vitro and in vivo. The potential mechanisms of these effects include inhibition of the production of inflammatory mediators [13, 19, 21], suppression of overactivated immune-responses, balancing the function of helper T cells (Th) and suppressor T cells (Ts), and inhibition of oxidative stress and Ca^2+^ overload. Some clinical trials have shown that TGP can markedly improve the quality of patients' lives and relieve the symptoms of JIA with lower incidences of side effects. However, no systematic review has yet been conducted.

The objective of this systematic review and meta-analysis is to pool the data from the included studies to examine the efficacy and safety of TGP compared with a control treatment or placebo in the treatment of JIA.

## 2. Materials and Methods

To ensure the accuracy of our systemic review and meta-analysis, we conducted our review in compliance with the Preferred Reporting Items for Systemic Reviews and Meta-Analyses (PRISMA) statement [22] as much as possible.

### 2.1. Eligibility Criteria

Randomized controlled trials (RCTs) that compared the effects of TGP against a control treatment (placebo, NSAIDs, DMARDs, or glucocorticoids) in people with JIA were included in this review, regardless of dates, language, blinding, or publication status. Patients must have received TGP alone or with other active drugs for a minimum of 4 weeks. JIA was diagnosed according to the 2000 International League of Associations for Rheumatology (ILAR) classification criteria [23]. TGP was defined as a water or ethanol extract of Radix Paeoniae Alba, which in this review included TGP capsules and TGP tablets. We excluded trials that used other herbal extracts, and studies without outcomes data were also eliminated.

### 2.2. Information Sources and Search

We searched the following electronic databases from their inception to September 30th 2015: PubMed, Cochrane Central Register of Controlled Trials, Cochrane Database of Systematic Reviews, ISI Web of knowledge, Chinese Biomedical Database (CBM), Chinese National Knowledge Infrastructure (CNKI), Wan Fang Database, and Chinese Science and Technique Journals Database (VIP). For the English databases, the search terms used were “Juvenile Idiopathic Arthritis” or “juvenile rheumatoid arthritis” or “JIA” or “JRA” or “Juvenile chronic arthritis” and “total glucosides of peony” or “total glucosides of Paeonia” or “TGP”. For the Chinese databases, the key search terms were as follows: “you nian te fa xing guan jie yan” or “er tong te fa xing guan jie yan” (the Chinese name of JIA) and “bai shao zong gan” (which means total glucosides of peony) or “Pa fu lin” (a type of total glucosides of peony tablet). All search strategies were restricted to human clinical trials. To obtain any other additional articles, we manually searched all the references of the relevant studies identified. We also contacted the authors for the details of unpublished and ongoing studies.

### 2.3. Study Selection

Two reviewers (Yongsong Cai and Qiling Yuan) first applied the eligibility criteria independently to screen the titles and abstracts of all the records and then sought the full text of studies meeting the inclusion criteria or that were ambiguous. The kappa value was used to measure the agreement between the two reviewers. Any inconsistencies were resolved by consensus or in consultation with a third reviewer (Peng Xu).

### 2.4. Data Collection Process

Two reviewers (Yongsong Cai and Qiling Yuan) independently extracted the data and entered it into a standard spreadsheet. The third reviewer (Jialin Zhu) verified the data accuracy. Any differences were resolved by consensus, and the authors were contacted to supplement any data lacking details.

### 2.5. Data Items

Information was extracted using structured data extraction tables including the following items: (1) study design; (2) patients' general data (including age, sex, diagnostic methods, subtype, and severity of JIA); (3) inclusion and exclusion criteria; (4) intervention and comparator (including dose of TGP, duration of follow-up, and type of comparator (placebo or other medicine); the duration of follow-up was defined as one of three terms: short-term (4–8 weeks), intermediate term (9–26 weeks), or long-term (>26 weeks), and the latest time point would be included if there were several time points in one term); (5) outcomes including changes of effectiveness (e.g., overall response rate, pain, tender joint count (TJC), swollen joint count (SJC), duration of morning stiffness (DMS), grip strength (GS), erythrocyte sedimentation rate (ESR), C-reactive protein (CRP), and adverse effects (AEs)). Missing essential information was sought by contacting the authors.

### 2.6. Quality Assessment for Individual Studies

Two authors (Yongsong Cai and Qiling Yuan) independently assessed the risk of bias in the individual studies with the Cochrane Handbook for Systematic Reviews of Interventions version 5.1.0 [35] according to the following aspects: random sequence generation, blinding of participants and personnel, blinding of outcome assessment, incomplete outcome data, selective reporting, and other sources of bias. Each of the criteria was judged using three categories: low (low risk of bias), high (high risk of bias), and unclear (lack of information or uncertainty about the potential for bias). Studies with more categories judged as “low” were identified as superior. Agreement between the two authors was assessed using the kappa value. Any inconsistencies were resolved by consensus and discussion.

### 2.7. Data Synthesis and Analysis

Data analyses were carried out with Stata/SE version 11 (STATA Corp., College Station, TX). We pooled the data with respect to the duration of follow-up (short term, intermediate term, and long term). For dichotomous outcomes, the odds ratios (ORs) and 95% confidence intervals (CIs) were used with the intention-to-treat (ITT) principles. For continuous data, the standard mean differences (SMDs) or the mean differences (MDs) and 95% CIs were calculated. SMDs were used when the same outcomes were assessed in a variety of ways, and if the outcomes were assessed on the same scale in all trials, the MDs were adopted. We used the *I* ^2^ statistic to quantify inconsistency across studies. A value of *I* ^2^ = 0% represents no observed heterogeneity, and larger values indicate increasing heterogeneity. When *I* ^2^ ≤ 25%, we reported the pooled results using a fixed-effect model; otherwise, a random-effects model was employed. If *I* ^2^ > 50%, we further explored the possible factors of variation. Egger's test was used to assess potential publication bias. A value of *p* < 0.05 signifies the existence of potential publication bias. A sensitivity analysis was performed to assess the robustness of the results for outcomes if applicable. A *p* value of less than 0.05 was considered significant for all analyses.

## 3. Result

### 3.1. Study Selection

**Figure 1:**
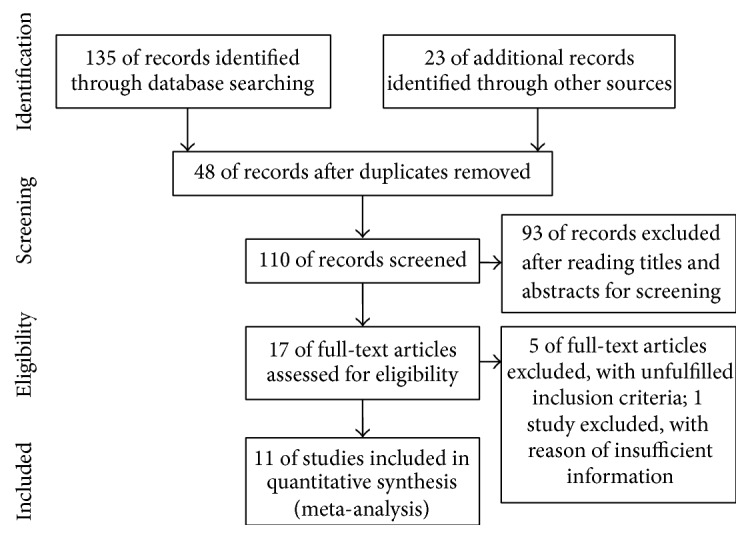
Flow diagram.

Our initial search identified 158 studies, of which 48 duplicate studies were removed. After reading the titles and abstracts, 93 articles were excluded. Next, we read the full text of the remaining 17 articles, and 5 of them were excluded because they did not meet the inclusion criteria. One study had insufficient information (i.e., did not report any data about outcomes); the author was contacted for additional data but did not respond. Finally, 11 references [24–34] with a total of 590 participants were included (TGP: 308, control: 282) (Figure 1). Agreement on study selection between the 2 reviewers had a favorable consistency (kappa value = 0.90).

### 3.2. Study Characteristics

Table 1 listed the characteristics of the included studies that were published from 2004 to 2012. Eleven RCTs including 590 participants were conducted in China and published in Chinese. The majority of the studies were conducted as single-center trials, with only one study [27] conducted as a multicenter trial. Overall, the age of participants in the nine trials [25–29, 31–34] ranged from 1.5 to 14 years, and the average age was 8.8 years, although Shi and Ding [24] and Tong and Shui [30] did not report the average age. Males accounted for 55% of the participants in the nine [25–27, 29–34] studies, and two studies [24, 28] did not mention the gender distribution. The majority of trials showed the composition of each subtype of JIA, although five trials did not [24, 25, 28, 30, 31]. Two of the trials [24, 26] including 99 participants compared TGP alone with Methotrexate (MTX) alone. Nine studies had more than two types of treatment in the intervention or control groups as follows: TGP plus DMARDs versus DMARDs alone in 7 trials [27–29, 31–34]; TGP plus NSAIDs and glucocorticoids versus a control of NSAIDs and glucocorticoids in one trial [25]; TGP plus NSAIDs and DMARDs versus a control of NSAIDs and DMARDs in one trial [30]. Different doses of TGP, which ranged from 30 mg/kg/d to 60 mg/kg/d, were used in these studies. Moderate doses of TGP were used in most of the studies, but one study [29] did not mention the doses used. The total daily TGP intake was divided into two to three doses. Treatment duration ranged from four weeks to 12 months. Most studies used response rate, ESR, CRP, TJC, SJC, DMS, and AEs for measuring outcomes. The response rate was defined as the proportion of the number of participants who reached an efficacy average index 30 (EAI30) in all studies except Zang [26] (EAI50) and Shi and Ding [24] (EAI20). The EAI30 was defined as an average minimum of 30% improvement from baseline in all the included outcomes. Only one study [34] used the ACR70 to evaluate the effect of TGP.

### 3.3. Risk of Bias within Studies

Table 2 shows the risk of bias across all studies. All the included clinical trials were associated with a high risk of bias. All the 11 included trials reported random sequence generation, but only one trial [33] provided the information regarding how randomization was performed. One trial [27] included the use of allocation concealment, and the processes used in the remaining studies were unclear. Blinding was unclear in all of the included studies. Agreement between the two authors for each aspect ranged from 72% to 100%, and the overall agreement was 90%.

### 3.4. Adverse Events

**Figure 2:**
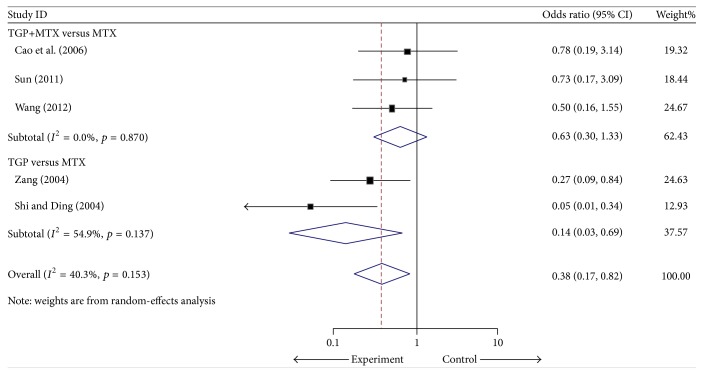
Forest plot of the random-effects model of the odds ratio (95% CI) in the AEs for TGP versus controls.

Five trials [24, 26, 27, 31, 34] including 157 intervention patients and 134 controls reported adverse events. The pooled OR (95% CI) in the TGP plus MTX versus MTX alone subgroup, the TGP alone versus MTX alone subgroup, and the overall group was 0.63 (0.30 to 1.33), 0.14 (0.03 to 0.69), and 0.38 (0.17 to 0.82), respectively, which indicated that the intervention group had a lower incidence of AEs in the TGP alone versus the MTX alone subgroup and the overall group (Figure 2 and Table 3). The main AEs in intervention were gastrointestinal events which included nausea, abdominal distention, anorexia, and diarrhea. In the control group, the main AEs included liver function abnormalities, leucopenia, rash, stomatitis, and anorexia. The majority of these events were mild to moderate in intensity and did not require treatment.

## 4. Intervention Efficacy

### 4.1. Cointervention versus Control

Nine trials compared a cointervention of TGP and DMARDs or NSAIDs or glucocorticoids with a control as follows: TGP plus DMARDs versus DMARDs alone in 7 trials [27–29, 31–34]; TGP plus NSAIDs and glucocorticoids versus a control of NSAIDs and glucocorticoids in one trial [25]; TGP plus NSAIDs and DMARDs versus a control of NSAIDs and DMARDs in one trial [30].

#### 4.1.1. The Overall Response Rate

**Figure 3:**
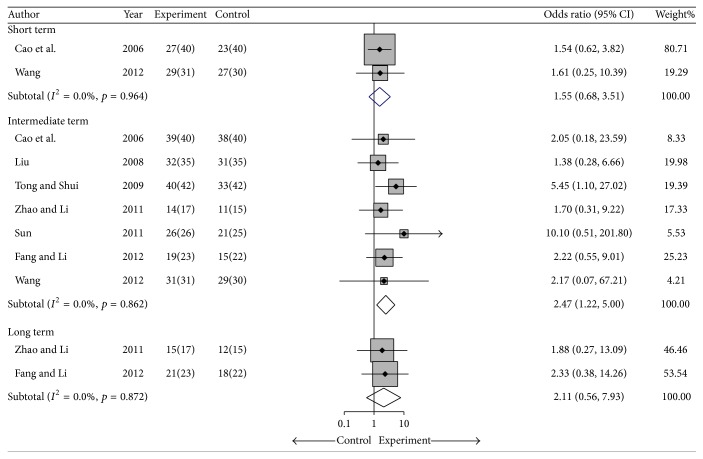
Forest plot of the fixed-effects model of the odds ratio (95% CI) in the overall response rate for cointervention versus controls in short term, intermediate term, and long term.

Seven studies (*n* = 423) compared a cointervention and a control group [27, 29–34]. The overall response rates were assessed at each treatment term, which included short term (4–8 weeks), intermediate term (9–26 weeks), and long term (>26 weeks), and the overall response rate at each term was as follows: OR = 1.55, 95% CI: 0.68 to 3.51; OR = 2.47, 95% CI: 1.22 to 5.0; and OR = 2.11, 95% CI: 0.56 to 7.93, respectively, with no significant heterogeneity (*I* ^2^ = 0%), which indicated that cointervention was favored in the overall response rate in the intermediate term (Figure 3). Sensitivity analysis was performed by using jackknife analysis in the intermediate term and, in most cases, removal of a study did not significantly change the result (OR ranged from 2.27 to 2.86, *p* < 0.03), except in the study by Tong (OR = 2.04; 95% CI: 0.93 to 4.47; *p* = 0.074) (Table 3 and Supplementary Materials available online at http://dx.doi.org/10.1155/2016/8292486). Egger's test indicated no significant publication bias (coefficient = 0.59; SE = 0.90; *t* = 0.65; *p* = 0.545) in the intermediate term (Supplementary Materials).

#### 4.1.2. ESR

**Figure 4:**
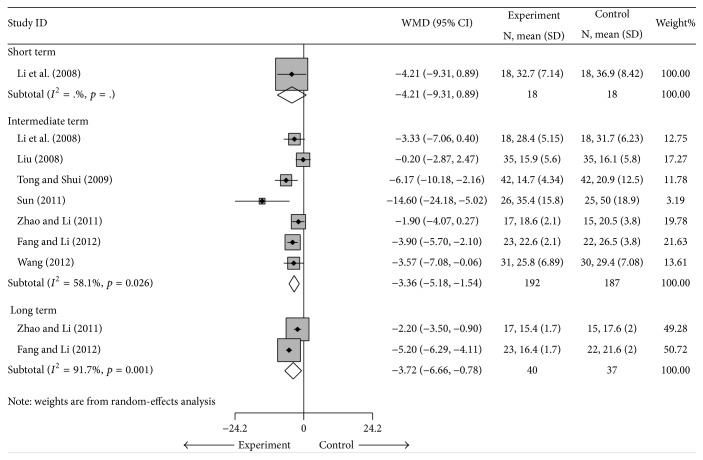
Forest plot of the random-effects model of the MD (95% CI) in the ESR for cointervention versus controls in short term, intermediate term, and long term.

Cointervention showed very large treatment effects for ESR compared to DMARDs alone [28, 29, 31–34] or DMARDs plus NSAIDs [30] (*n* = 379). The pooled MD for ESR was −4.21 mm/h (95% CI: −9.31 to 0.89 mm/h) in the short term, −3.36 mm/h (95% CI: −5.18 to −1.54 mm/h) in the intermediate term, and −3.72 mm/h (95% CI: −6.66 to −0.78 mm/h) in the long term. That is, the ESR in the cointervention groups was 3.36 mm/h lower than the ESR of the control groups in the intermediate term and 3.72 mm/h lower than the control groups at the long term, but heterogeneity existed in the intermediate term (*I* ^2^ = 58.1%, *p* = 0.026) and long term (*I* ^2^ = 91.7%, *p* = 0.001) (Figure 4). The source of the heterogeneity in the intermediate term was not apparent. A sensitivity analysis was performed by pooling the 6 studies [28–30, 32–34] with cointerventions versus DMARDs in the intermediate term, and the treatment effect was still significant, with an MD of −2.93 mm/h (95% CI: −4.92 to −1.44 mm/h) (Table 3 and Supplementary Materials). Egger's test indicated no significant publication bias (coefficient = −2.03; SE = 1.38; *t* = −1.47; *p* = 0.203) in the intermediate term (Supplementary Materials).

#### 4.1.3. CRP

**Figure 5:**
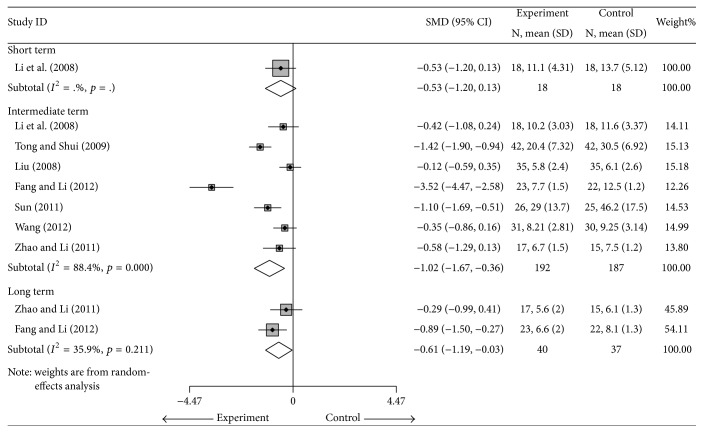
Forest plot of the random-effects model of the SMD (95% CI) in the CRP for cointervention versus controls in short term, intermediate term, and long term.

Seven studies [28–34] with a total of 379 participants compared the CRP between the cointervention groups and the groups using DMARDs alone [28, 29, 31–34] or DMARDs plus NSAIDs [30]. In the random-effects model, the SMDs in the short term, intermediate term, and long term were −0.53 (95% CI: −1.2 to 0.13), −1.02 (95% CI: −1.67 to −0.36), and −0.61 (95% CI: −1.19 to −0.03), respectively. That is, patients in the cointervention groups had a 1.02-unit significantly lower CRP in intermediate term and 0.61-unit lower CRP in long term than the patients in the control group (Figure 5). There was high heterogeneity in the intermediate term (*I* ^2^ = 88.4%). The source of this heterogeneity was unclear, but when two studies were removed [30, 33], the SMD was −0.49 (95% CI: −0.82 to −0.15, *I* ^2^ = 41.3%) (Table 3 and Supplementary Materials). In the sensitivity analysis limited to the 6 studies [28, 29, 31–34] with cointervention versus DMARDs in intermediate term, the SMD was −0.95 (95% CI: −1.7 to −0.2) (Table 3 and Supplementary Materials). No significant publication bias was found by Egger's test (coefficient = −7.5; SE = 4.66; *t* = −1.62; *p* = 0.166) in intermediate term (Supplementary Materials).

#### 4.1.4. DMS

**Figure 6:**
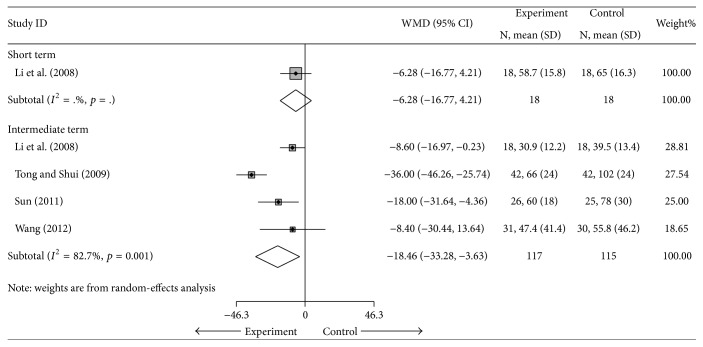
Forest plot of the random-effects model of the MD (95% CI) in the DMS for cointervention versus controls in short term and intermediate term.

Four studies [28, 30, 31, 34] (*n* = 232) compared cointervention with DMARDs alone [28, 31, 34] or DMARDs plus NSAIDs [30] for the outcome of DMS and had a pooled MD of −6.28 min (95% CI, −16.77 to 4.21 min) in short term and −18.46 min (95% CI, −33.28 to −3.63 min) in intermediate term (Figure 6). The results indicated that cointervention had a shorter DMS than the control group in intermediate term. However, the results were heterogenous (*I* ^2^ = 82.7%). Sensitivity analysis was performed using the 3 studies [28, 31, 34] with cointervention versus DMARDs alone; the MD was −10.91 min (95% CI: −17.69 to −4.21) and *I* ^2^ = 0%, which indicated that Tong [30] was the main source of this heterogeneity (Table 3 and Supplementary Materials). There was no evidence of publication bias (coefficient = −0.07, SE = 4.7, *t* = −0.01, *p* = 0.99) (Supplementary Materials).

#### 4.1.5. TJC

Two studies [28, 31] with a total of 87 patients compared TJC between the cointervention group and DMARDs alone. The MD was −2.19 (95% CI: −4.53 to 0.15) in short term and −2.99 (95% CI: −5.82 to −0.16) in intermediate term, meaning that the cointervention group had less TJC than the DMARD only group in intermediate term (Table 3 and Supplementary Materials).

#### 4.1.6. SJC

Two studies [28, 31] compared SJC between the cointervention group and the group using DMARDs alone (*n* = 87). No effect was found in short term (MD = −2.41; 95% CI: −4.97 to 0.152) and intermediate term (MD = −3.05; 95% CI: −6.97 to 0.87) (Table 3 and Supplementary Materials).

#### 4.1.7. Autoantibodies

Two studies (*n* = 93) [25, 34] compared IgG and IgA, and the pooled MDs were −0.90 (95% CI: −3.39 to 1.59) and −0.48 (95% CI: −1.05 to 0.10), respectively (Table 3 and Supplementary Materials).

#### 4.1.8. Other

Tong and Shui [30] compared GS in intermediate term and the pooled MD was 1.83 kg (95% CI: 0.68 to 2.98; *p* = 0.002); Liu [29] compared the prednisone dosage and prednisone treatment duration and the pooled MD was −2.40 mg/d (95% CI: −3.75 to −1.05; *p* = 0.001) and −56 d (95% CI: −75.12 to −36.88; *p* < 0.001), respectively. Only one study [34] described the response rate of ACR70 (OR = 3.61; 95% CI: 1.09 to 11.94; *p* = 0.04) and Juvenile Arthritis Disease Activity Score (JADAS) (MD = −1.75; 95% CI: −2.24 to −1.26; *p* < 0.001) (Table 3 and Supplementary Materials).

### 4.2. TGP Compared with DMARDs Alone

#### 4.2.1. The Overall Response Rate

Two studies [24, 26] with a total of 99 patients were pooled to compare TGP alone with MTX alone. The OR was 0.60 (95% CI: 0.12 to 3.03) in short term and 1.26 (95% CI: 0.29 to 5.47) in intermediate term with no significant heterogeneity (*I* ^2^ = 0%). That is, patients receiving TGP alone had a better response rate than patients who received MTX alone, but this finding did not reach statistical significance (*p* = 0.761 in intermediate term) (Table 3 and Supplementary Materials).

Only one study [26] described the result of continuous data in TGP versus MTX alone group, and the duration of treatment was 6 months. A significant difference was identified between TGP and the MTX alone group in terms of the pain score (*p* = 0.010; MD = −0.30; 95% CI: −0.53 to −0.07), dose of prednisone (*p* = 0.003; MD = −2.40 mg/d; 95% CI: −3.96 to −0.84), and course of prednisone (*p* < 0.001; MD = −56 d; 95% CI: −77.71 to −34.29), signifying that the TGP group could shorten the course of prednisone, alleviate pain, and decrease the dose of prednisone. However, no effects were found in DMS (*p* = 0.316; MD = −3.36 min; 95% CI: −9.76 to 3.16), ESR (*p* = 0.169; MD = −4.40 mm/h; 95% CI: −10.67 to 1.87), or CRP (*p* = 0.651; MD = −1.6 mg/L; 95% CI: −8.53 to 5.34) (Table 3).

## 5. Discussion

To the best of our knowledge, this is the first systematic review and meta-analysis evaluating the efficacy and safety of TGP for the treatment of JIA. We pooled the results from 11 studies including 590 participants. To minimize the heterogeneity, three subgroups were set according to the treatment duration. The relevant clinical outcomes were studied, including ESR, CRP, DMS, SJC, TJC, autoantibodies, and treatment response rates, as well as other relevant clinical outcomes (i.e., GS, ACR70, pain score, and DAS). Overall, the results of this meta-analysis suggested favorable effects of TGP plus DMARDs or NSAIDs in patients with JIA in intermediate term (9–26 W), and the overall incidence of AEs was lower in intervention group. However, statistical significance was not attained in most of the results in short term and long term, especially in the TGP alone versus DMARD alone groups, which might be explained by the lack of sufficient studies included. In addition, the overall methodological quality was low. Therefore, caution should be exercised in interpreting these positive results.

In this meta-analysis, TGP combined with DMARDs or NSAIDs showed better effects on the ESR, CRP, DMS, and treatment response rates. Another highlight of our study was the finding that the intervention groups had a lower incidence of AEs (RR = 0.58, 95% CI: 0.38 to 0.91), particularly the incidence of hepatotoxicity (2/308 versus 6/282, RR = 0.31, 95% CI: 0.06 to 1.52). Some findings might explain the potential therapeutic effect of TGP in the treatment of JIA. Preclinical studies have shown that TGP displays immune-regulatory, anti-inflammatory, and hepatoprotective activities. In experimental arthritis rats, intragastric administration of TGP (25−100 mg/kg/d) for 7–14 days was demonstrated to notably diminish the severity of hind paw swelling and the scores of polyarthritis in a dose-dependent manner [7–10, 12, 36, 37]. The synovial infiltration of lymphocytes [8, 9, 12], hypertrophy of synovial membranes, and the formation of new blood vessels and pannus were also significantly inhibited by TGP [8, 9]. In vitro, paeoniflorin could induce apoptosis in murine T lymphocytes and Jurkat human T cell leukemia cells [11]. The most exciting effect of TGP was its hepatoprotective activity. Paeoniflorin has been shown to protect mice against* Schistosoma japonicum* egg-induced hepatic fibrosis by interfering with the IL-13 signaling molecule and decreasing the level of IL-13 [38, 39]. Other studies have also shown that paeoniflorin could protect mice against* Bacillus* Calmette-Guerin or lipopolysaccharide-induced liver injury [14, 40], which might be associated with the effects of inhibition of IL-1*β* and TNF-*α* release and the promotion of IL-10 production [21]. Recent research has also suggested that TGP could reduce the hepatotoxicity caused by combination treatment with Methotrexate and Leflunomide for active rheumatoid arthritis [18]. All the data supported the potential therapeutic effect of TGP in the treatment of JIA.

Three meta-analyses have been published on the efficacy and safety of TGP for the treatment of RA [41–43], but all of them have been published in Chinese. Among the three meta-analyses, two of them [41, 42] drew positive conclusions with the OR of TGP versus the control group for the overall response rates, which were 1.13 (95% CI: 1.04 to 1.24) and 2.65 (95% CI: 1.51 to 4.65), respectively. One trial [43] drew negative conclusions with the OR for the overall response rate (RR = 3.41, 95% CI: 0.77 to 15.10). The incidence of AEs in all the trials ranged from 0.41 to 0.66, and the incidence of hepatotoxicity ranged from 0.19 to 0.41. Most of the included studies indicated that TGP demonstrates beneficial effects on the symptoms of RA with a lower incidence of AEs, especially the incidence of hepatotoxicity. However, all of them also found that the incidence of diarrhea was higher in patients treated with TGP than in patients who were not treated with TGP. Our study resulted in similar conclusions. Considering that the treated subjects are children, the incidence of diarrhea should be noted when using TGP for the treatment of JIA.

The results of our meta-analysis should be treated with caution for several reasons. First, all the included trials were conducted in China, which was a potential selection bias, and might influence the application of TGP for patients in other countries. Second, the selected trials did not report ethical approval, which may weaken the validity of the results. Third, the methodological quality of the included studies was poor; while all of the selected studies were described as randomized controlled trials, only one trial [33] provided the information regarding how randomization was performed. Only one trial mentioned allocation concealment [27], and the blinding was unclear in all of the included studies. Fourth, in our study, we pooled the results without regard for the subtype of JIA, because treatment according to subtypes was not used in most of the included trials, which may affect the applicability for particular subtypes. Fifth, small sample sizes, insufficient numbers of included trials in each subgroup, and extensive heterogeneity in some results weaken the overall results of the review. Sixth, except for CRP, there was no other inflammatory factor, such as TNF-*α* or PGE2, which may weaken the persuasive. Lastly, the outcome measures used were inadequate, as only one trial used the American College of Rheumatology Pediatric (ACR Pedi) response criteria [34] and the Juvenile Arthritis Disease Activity Score (JADAS), and none of the included studies used tools for the assessment of quality of life, which is very important for evaluating the therapeutic effect, such as the Childhood Health Assessment Questionnaire (CHAQ), Child Health Questionnaire (CHQ), and Pediatric Quality of Life (PedsQL) [5]. All of these limitations may influence the application of TGP for treatment of JIA and weaken the validity of the results.

To effectively compare treatment options in different countries, future clinical RCTs should follow standardized and validated outcome measures and use accepted standards of trial design and reporting (CONSORT).

In conclusion, TGP is safe and efficacious for the treatment of JIA with few AEs. TGP, which has similar effects on nonbiologic DMARDs, is a special type of nonbiologic DMARD for the treatment of JIA.

## Supplementary Material

The outcomes of the present review were grouped into this file. Egger's publication bias plots identifying publication bias for all studied outcomes and all the forest plots of meta-analyses and sensitivity-analyses and subgroup-analyses were included in this file.

## Figures and Tables

**Table 1:** Characteristic of included studies.

Study	Case number (I/C)	Age (years)	Sex (male/female)	Subtype of JIA	Intervention (I)	Control (C)	Outcomes	Duration
Shi and Ding (2004) [24]	18/13	Unclear	Unclear	Unclear	TGP 0.9 g/d, oral.	MTX 10 mg/m^2^/w, oral	Overall response rate, AE	6 W, 24 W

Tang et al. (2004) [25]	16/16	9.5	17/15	Unclear	TGP 30–60 mg/kg/d, oral. Naproxen 10–12 mg/kg/d, prednisone 1-2 mg/kg/d, oral.	Naproxen 10–12 mg/kg/d, prednisone 1-2 mg/kg/d, oral	Overall response rate, autoantibody	2 M

Zang (2004) [26]	42/26	8.6 (3–14)	32/36	Poly 34, Pauci 18, and systemic 16	TGP 0.03–0.06 g/kg/d, oral.	MTX 0.2–0.4 mg/kg/w, oral	Overall response rate, ESR, CRP, DMS, prednisone dosage, prednisone treatment duration, pain score, and AE	6 M

Cao et al. (2006) [27]	40/40	9.5 ± 3.0	52/28	Pauci 25, poly 11, and systemic 44	TGP 0.018 g/kg, twice a day orally; MTX 10 mg/m^2^ and folic acid 5 mg, once a week orally.	MTX 10 mg/m^2^ and folic acid 5 mg, once a week orally	Overall response rate, AE	8 W, 24 W

Li et al. (2008) [28]	18/18	7.1 ± 2.6	Unclear	Unclear	TGP 0.015 g/kg, twice a day orally, MTX 10 mg/m^2^, and folic acid 5 mg, once a week orally.	MTX 10 mg/m^2^ and folic acid 5 mg, once a week orally	ESR, CRP, DMS, TJC, and SJC	8 W, 12 W

Liu (2008) [29]	35/35	8.6	41/29	Systemic 70	TGP + MTX, oral dose (unclear).	MTX, oral dose (unclear)	Overall response rate, CRP, ESR, prednisone dosage, and prednisone treatment duration	6 M

Tong and Shui (2009) [30]	42/42	3–15	38/46	Unclear	TGP 15–30 mg/kg, twice a day orally, Lef 0.2–0.4 mg/kg, and Mel 5–7.5 mg, once a week orally.	Lef 0.2–0.4 mg/kg, Mel 5–7.5 mg, once a week orally	Overall response rate, CRP, ESR, DMS, and GS	6 M

Sun (2011) [31]	26/25	7.8 (1.5–14)	24/27	Unclear	TGP 30–60 mg/kg/d, oral. MTX 10 mg/m^2^/w, oral.	MTX 10 mg/m^2^/w, oral	Overall response rate, CRP, ESR, DMS, TJC, SJC, and AE	26 W

Zhao and Li (2011) [32]	17/15	8.1 ± 2.5	18/14	Poly 15, Pauci 10, and other 7	TGP 0.3 g, twice a day orally, MTX 10 mg/m^2^, once a week orally.	MTX 10 mg/m^2^ once a week orally	Overall response rate, CRP, and ESR	6 M, 12 M

Fang and Li (2012) [33]	23/22	8.1 ± 2.5	26/19	Systemic 45	TGP 0.3 g, twice a day orally, MTX 10 mg/m^2^, once a week orally.	MTX 10 mg/m^2^ once a week orally	Overall response rate, CRP, and ESR	6 M, 12 M

Wang (2012) [34]	31/30	10.6 ± 2.9	38/23	Pauci 46, poly 13, and systemic 2	TGP 0.6 g, 3 times a day orally, MTX 0.25–0.8 mg/kg, once a week orally.	MTX 0.25–0.8 mg/kg, once a week orally	Overall response rate, CRP, ESR, DMS, autoantibody, ACR70, JADAS, and AE	8 W, 12 W

Pauci: pauciarticular; poly: polyarticular; Lef: Leflunomide; Mel: meloxicam; DMS: duration of morning stiffness; TGP: total glucosides of peony; MTX: Methotrexate; TJC: tender joint count; SJC: swollen joint count; GS: grip strength; AE: adverse effect; CRP: C-reactive protein; ESR: erythrocyte sedimentation rate; JADAS: Juvenile Arthritis Disease Activity Score.

**Table 2:** Methodological quality of included trials.

Author	Random sequence generation	Allocation concealment	Blinding of participants and personnel	Blinding of outcome assessment	Incomplete outcome data	Selective reporting	Other sources of bias
Shi and Ding (2004) [24]	U	U	H	U	H	H	H
Tang et al. (2004) [25]	U	U	H	U	U	L	U
Zang (2004) [26]	U	U	U	U	L	L	U
Cao et al. (2006) [27]	U	L	U	U	U	L	U
Li et al. (2008) [28]	U	U	U	U	H	H	U
Liu (2008) [29]	U	U	H	U	H	U	U
Tong and Shui (2009) [30]	U	U	H	U	H	U	U
Sun (2011) [31]	U	U	U	U	L	L	U
Zhao and Li (2011) [32]	U	U	H	U	U	H	U
Fang and Li (2012) [33]	L	U	H	U	U	H	U
Wang (2012) [34]	U	U	H	U	L	L	U

U: unclear; H: high risk; L: low risk.

**Table 3:** Subgroup and sensitivity analysis.

Treatment	Outcomes	Subgroup	Number of studies and participants	Effect size (SMD, MD, OR, or RR)	Effect size, *p* value	Heterogeneity, *I* ^2^ (%)	Heterogeneity, *p* value
Cointervention versus control	Overall response rate		7,423				
		Short term	2,141	OR = 1.55, 95% CIs, 0.68 to 3.51	0.295	0	0.963
		Intermediate term	7,423	OR = 2.47, 95 CIs, 1.22 to 5.0	0.012	0	0.862
		Long term	2,77	OR = 2.11, 95% CIs, 0.56 to 7.93	0.27	0	0.872
		Intermediate term	6,339^#^	OR = 2.04, 95% CI 0.93 to 4.47	0.074	0	0.925
	ESR		7,379				
		Short term	1,36	MD = −4.21 mm/h, 95% CI, −9.31 to 0.89 mm/h	0.106	—	—
		Intermediate term	7,379	MD = −3.36 mm/h, 95% CI, −5.18 to −1.54 mm/h	<0.001	58.1	0.026
		Long term	2,77	MD = −3.72 mm/h, 95% CI, −6.66 to −0.78 mm/h	0.013	91.7	0.001
		Intermediate term	6,328^#^	MD = −2.93 mm/h, 95% CI, −4.92 to −1.44 mm/h	<0.001	42.3	0.123
	CRP		7,379				
		Short term	1,36	SMD = −0.53, 95% CI, −1.2 to 0.13	0.117	—	—
		Intermediate term	7,379	SMD = −1.02, 95% CI, −1.67 to −0.36	0.002	88.4	<0.001
		Long term	2,77	SMD = −0.61, 95% CI, −1.19 to −0.03	0.038	35.9	0.211
		Intermediate term	5,250^#^	SMD = −0.49, 95% CI, −0.82 to −0.15	0.005	41.3	0.146
		Intermediate term	6,295^#^	SMD = −0.95, 95% CI, −1.70 to −0.20	0.013	88.7	<0.001
	DMS		4,232				
		Short term	1,36	MD = −6.28 min, 95% CI, −16.77 to 4.21 min	0.241	—	—
		Intermediate term	4,232	MD = −18.46 min, 95% CI, −33.28 to −3.63 min	0.015	82.7	0.001
		Intermediate term	3,148^#^	MD = −10.91 min, 95% CI, −17.69 to −4.12 min	0.002	0	0.502
	TJC		2,87				
		Short term	1,36	MD = −2.19, 95% CI: −4.53 to 0.15	0.066	—	—
		Intermediate term	2,87	MD = −2.99, 95% CI: −5.82 to −0.16	0.038	79.9	0.026
	SJC		2,87				
		Short term	1,36	MD = −2.41, 95% CI: −4.97 to 0.152	0.065	—	—
		Intermediate term	2,87	MD = −3.05, 95% CI: −6.97 to 0.87	0.127	92.8	<0.001
	IgG	Intermediate term	2,87	MD = −0.90 g/L, 95% CI: −3.39 to 1.59 g/L	0.478	80.7	0.023
	IgA	Intermediate term	2,87	MD = −0.48 g/L, 95% CI: −1.05 to 0.10 g/L	0.103	85.2	0.009
	GS	Intermediate term	1,84	MD = 1.83 kg, 95% CI: 0.68 to 2.98 Kg	0.002	—	—
	Prednisone dosage	Intermediate term	1,70	MD = −2.40 mg/d, 95% CI: −3.75 to −1.05 mg/d	0.001	—	—
	Prednisone treatment duration	Intermediate term	1,70	MD = −56 d, 95% CI: −75.12 to −36.88 d	*p* < 0.001	—	—
	ACR70	Intermediate term	1,61	OR = 3.61; 95% CI: 1.09 to 11.94	0.035	—	—
	JADAS	Intermediate term	1,61	MD = −1.75; 95% CI: −2.24 to −1.26	*p* < 0.001	—	—

TGP alone versus MTX alone	Overall response rate		2,99				
		Short term	1,31	OR = 0.60, 95% CI: 0.12 to 3.03	0.537	—	—
		Intermediate term	2,99	OR = 1.26, 95% CI: 0.29 to 5.47	0.761	0	0.963
	DMS	Intermediate term	1,68	MD = −3.36 min, 95% CI: −9.76 to 3.16 min	*p* = 0.316	—	—
	Pain score	Intermediate term	1,68	MD = −0.30; 95% CI: −0.53 to −0.07	*p* = 0.010	—	—
	Doses of prednisone	Intermediate term	1,68	MD = −2.40 mg/d, 95% CI: −3.96 to −0.84 mg/d	*p* = 0.003	—	—
	Course of prednisone	Intermediate term	1,68	MD = −56 d, 95% CI: −77.71 to −34.29 d	*p* < 0.001	—	—
	ESR	Intermediate term	1,68	MD = −4.40 mm/h; 95% CI: −10.67 to 1.87 mm/h	*p* = 0.169	—	—
	CRP	Intermediate term	1,68	MD = −1.6 mg/L; 95% CI: −8.53 to 5.34 mg/L	*p* = 0.651	—	—
	AEs	Overall	5,291	OR = 0.38, 95% CI: 0.17 to 0.82	0.014	40.3	0.153
		Cointervention versus control	3,192	OR = 0.63, 95% CI: 0.30, 1.33	0.226	0	0.870
		TGP alone versus MTX alone	2,99	OR = 0.14, 95% CI: 0.03, 0.69	0.016	54.9	0.137

^#^Sensitivity analysis after some studies were dropped out. DMS, duration of morning stiffness; TGP, total glucosides of peony; MTX, Methotrexate; TJC, tender joint count; SJC, swollen joint count; GS, grip strength; AE, adverse effect; CRP, C-reactive protein; ESR, erythrocyte sedimentation rate; JADAS, Juvenile Arthritis Disease Activity Score.
