# Supplementary material for: Assessment of the Therapeutic Effect of Total Glucosides of Peony for Juvenile Idiopathic Arthritis: A Systematic Review and Meta-Analysis
Source: Evid Based Complement Alternat Med. 2016 Jul 25;2016:8292486. doi: 10.1155/2016/8292486 (PMC4976154; doi:10.1155/2016/8292486)
Supplement: Supplementary file 1 — The outcomes of the present review were grouped into this file. Egger's publication bias plots identifying publication bias for all studied outcomes and all the forest plots of meta-analyses and sensitivity-analyses and subgroup-analyses were included in this file. [file 8292486.f1.zip › 8292486.f1.docx]

S1. Autoantibody
